# Inflammatory cytokines and their potential role in Sjogren’s syndrome risk: insights from a mendelian randomization study

**DOI:** 10.1186/s42358-024-00354-2

**Published:** 2024-02-16

**Authors:** Wenbin Shi, Yuli Xu, Anan Zhang, Xiqun Jia, Shuhua Liu, Ziyang Hu

**Affiliations:** 1 Department of Stomatology, Shenzhen Longhua District Central Hospital, Guanlan Avenue 187, Shenzhen City, Guangdong Province 518110 P. R. China; 2 Department of Neonatalogy, Shenzhen Longhua District Central Hospital, Guanlan Avenue 187, Shenzhen City, Guangdong Province 518110 P. R. China; 3 Department of Pediatrics, Shenzhen Longhua District Central Hospital, Guanlan Avenue 187, Guangdong Province Shenzhen Cit, 518110 P. R. China

**Keywords:** Sjogren’s Syndrome, Mendelian randomization, Cytokine, Genome-wide Association study, SNPs

## Abstract

**Aim:**

This study aimed to investigate the causal impact of inflammatory cytokines on Sjogren’s Syndrome (SS) and to identify potential biomarkers for SS clinical management using Mendelian Randomization (MR).

**Materials and methods:**

Leveraging GWAS summary data of inflammatory cytokines and SS, we executed the first two-sample MR analysis. Genetic variants from prior GWASs associated with circulating inflammatory cytokines served as instrumental variables (IVs). Data regarding cytokines were analyzed using the Olink Target-96 Inflammation panel, synthesizing data from 14,824 participants. GWAS summary statistics for SS were procured from the UK Biobank, focusing on samples of European ancestry. To discern the causal relationship between inflammatory cytokines and SS, several MR methodologies, including inverse variance weighted (IVW) and MR-Egger regression, were applied.

**Results:**

After rigorous IV quality control, 91 cytokines were incorporated into the MR analysis. The IVW analysis identified 8 cytokines with a positive association to SS: Axin-1 (OR 2.56, 95% CI 1.07–6.10), T-cell surface glycoprotein CD5 (OR 1.81, 95% CI 1.08–3.02), CUDP1 (OR 1.61, 95% CI 1.00-2.58), CXCL10 (OR 1.92, 95% CI 1.25–2.95), IL-4 (OR 2.18, 95% CI 1.22–3.91), IL-7 (OR 2.35, 95% CI 1.27–4.33), MCP-2 (OR 1.27, 95% CI 1.05–1.54), and TNFRSF9 (OR 1.83, 95% CI 1.03–3.24), suggesting their potential in increasing SS risk.

**Conclusion:**

Our study conducted through MR, identified various inflammatory cytokines associated with SS risk, validating some previous research results and offering some new potential biomarkers for SS. However, these findings necessitate further research for validation and exploration of their precise role in the onset and progression of SS.

**Supplementary Information:**

The online version contains supplementary material available at 10.1186/s42358-024-00354-2.

## Introduction

Sjogren’s Syndrome (SS), a prevalent autoimmune epithelitis, primarily targets the lacrimal and salivary glands, resulting in conditions such as xerophthalmia and xerostomia [1, 2]. The disease manifests either as primary SS, a standalone condition, or secondary SS, where it associates with other connective tissue diseases [2]. The pathological hallmark of SS is the infiltration of lymphocytes, mainly CD4 + T cells, into the secretory organs [3]. This disease has been implicated in a range of systemic symptoms, such as fever, arthralgia, and long-term fatigue, further complicating its clinical presentation [4].

Understanding the pathogenesis of SS is a complex undertaking, involving a myriad of cellular and molecular players [2, 5]. Among these, cytokines have emerged as critical factors in the ongoing debate about the disease’s etiology [6]. Initially, the immune response in SS was largely attributed to T cells, particularly the T helper cells (Th), categorized into Th1 and Th2 based on their cytokine profile [7]. However, the advent of newer subsets like Th17 cells and the acknowledgment of cytokine-producing B cells have added layers of complexity to this already intricate landscape [8, 9]. In the early stages of SS, lymphocytes infiltrate the affected glands, setting the stage for an inflammatory cascade [10]. Cytokines such as interleukin (IL)-17 produced by Th17 cells and others like interferon (IFN)-γ play a central role in mediating this inflammation. As the disease progresses, the balance of these cytokines can shift, influencing the severity and the type of symptoms experienced by the patient [11]. Despite strides in understanding SS, numerous enigmas endure. The precise role of cytokines in manifesting SS’s clinical symptoms, and the extent to which they underpin systemic symptoms, remains shrouded [12]. The inadequacy of T-cell-centric therapies has cast doubts on the predominant role of T cells in SS, thereby pivoting the spotlight towards B cells and their cytokine profiles [13]. Persistent research voids, fueled by inconsistent findings often stemming from constrained sample sizes or study design inadequacies, beckon further inquiry to unfurl the mystifying immunological tapestry of SS.

Mendelian Randomization (MR) is emerging as a powerful analytical tool for making sense of the intricate web of factors that contribute to diseases. Utilizing genetic variations associated with exposures, MR can act like a “genetic detective” to infer potential causal relationships between these exposures and observed health outcomes [14, 15]. It is often compared to a “natural” randomized controlled trial (RCT), as it exploits the random distribution of alleles during gamete production [16, 17]. This process is like Mother Nature’s own version of a blind draw, effectively minimizing confounding variables and biases, including those resulting from reverse causality [18]. In recent years, MR studies have shed new light on the complex interplay between autoimmune diseases and neurodegenerative conditions, such as Alzheimer’s Disease [19]. Specifically, this research has suggested that liability to autoimmune diseases like multiple sclerosis and SS may be associated with Alzheimer’s Disease, although the mechanisms behind these associations warrant further exploration. At the same time, MR studies have looked into other factors related to SS. One study focused on how gut bacteria could affect the risk of getting SS [20]. Some bacteria were found to increase the risk, while others reduced it. Another study checked if vitamin D levels had any effect on SS but didn’t find a clear link [21].

In this study, we performed the first two-sample MR analysis of the Genome-Wide Association Studies (GWASs) summary data containing inflammatory cytokines and SS, revealed the causal impact of inflammatory cytokines on SS, provided new biomarkers for the clinical management of SS.

## Materials and methods

### Study design

**Fig. 1:**
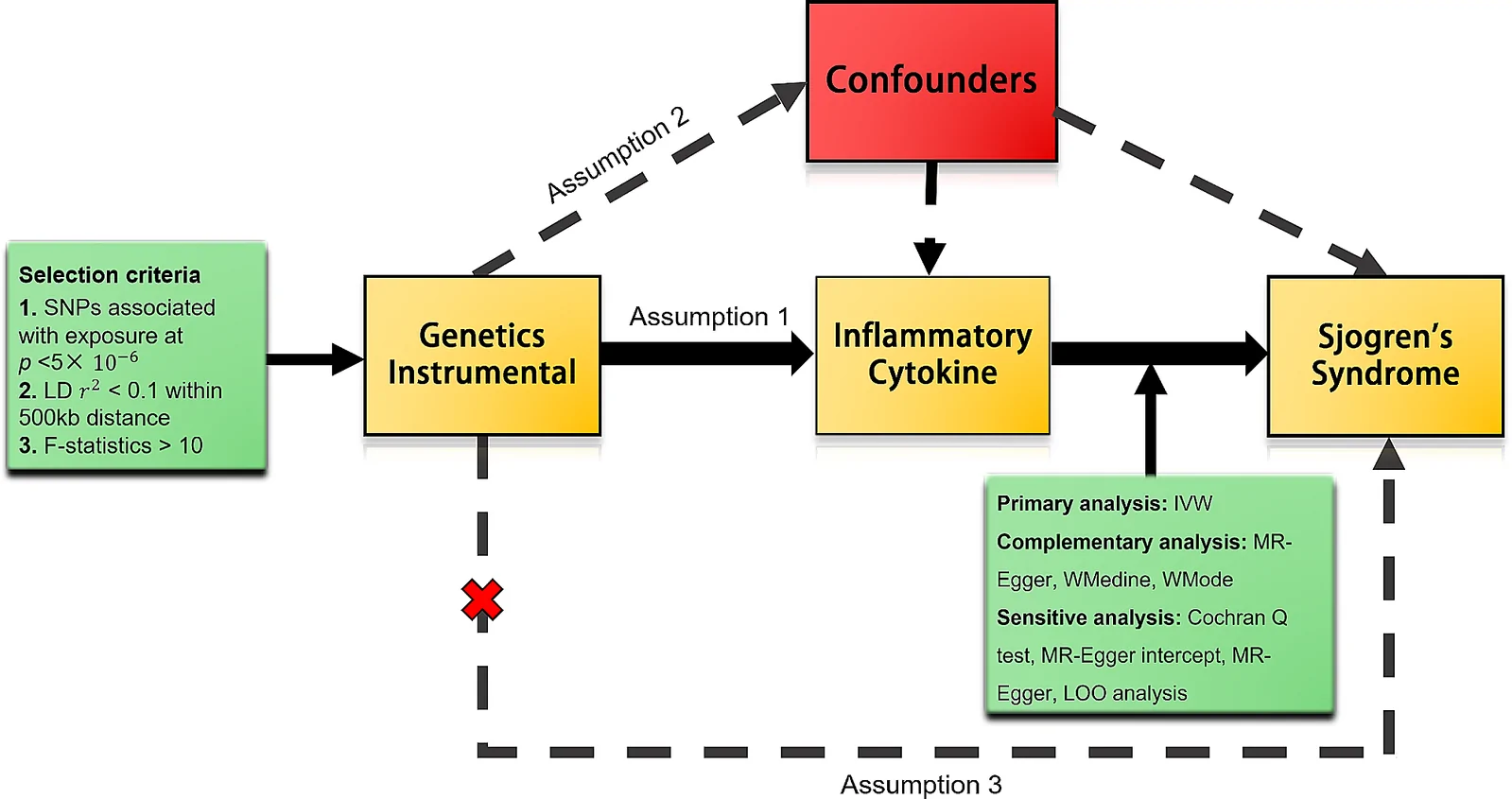
Diagram of the MR analysis. Assumption 1, genetic instruments are strongly associated with the exposures of interest; Assumption 2, genetic instruments are independent of confounding factors; Assumption 3, genetic instruments are not associated with outcome and affect outcome only via exposures. IVW, inverse variance weighted; LD, linkage disequilibrium; LOO analysis, leave-one-out analysis; WMedine, weighted medine; SNPs, single nucleotide polymorphisms; WM, weighted mode

The MR study conducted adhered to three core instrumental variable (IV) assumptions. (1) the genetic variants selected must exhibit a correlation with the exposure. (2) the chosen variants should remain free from any confounding factors. (3) the variants should only influence the outcome through the exposure (Fig. 1). Data summaries pertaining to circulating inflammatory cytokines and SS were acquired from publicly accessible GWASs, with a primary focus on cohorts of European descent as cited by reference [8]. The methodology of this MR study was aligned with the guidelines stipulated by Strengthening the Reporting of Observational Studies in Epidemiology using MR (STROBE-MR).

### Circulating inflammatory cytokines data source

GWAS summary statistics for circulating inflammatory cytokines were obtained from an up-to-date research launched by the SCALLOP Consortium [22]. The study was designed to explore inflammatory responses resulting in tissue damage and are central to the pathogenesis of multiple diseases, including sepsis, autoimmunity and atherothrombosis. Cytokines were analyzed using the Olink Target-96 Inflammation panel, with 91 proteins included due to BDNF issues [22]. Key or potential cytokines such as IL-4 [23], IL-6 [24], IL-7 [25], IL-10 [26], IL-12 [27], IL-15 [28], IL-17 [29], IL-22 [30], IL-33 [31], C-X-C motif chemokine 10 (CXCL10) [32], etc. for Sjogren’s disease research were included in the study (Table S1). The data was generated at Olink’s labs in Uppsala. Genotyping used SNP arrays, with imputation via 1000 Genomes or HRC panel. A GWAS analysis was employed for each protein in the pQTL mapping. The meta-analysis synthesized data from 14,824 participants, setting a statistical significance at *P* ≤ 5 × 10^− 10^ [22]. Results were compared to the ARISTOTLE study [33, 34]. pQTLs, reflecting protein abundance associations, were clearly defined. Protein variance was computed through a formula, and conditional analysis utilized GCTA [22].

### SS data source

GWAS summary statistics for SS were obtained from the UK Biobank [35]. The study involved approximately 500,000 participants aged 40–69, with extensive phenotypic information recorded. Genotyping was executed with the Affymetrix UK BiLEVE Axiom array for the first 50,000 and the Affymetrix UK Biobank Axiom for the rest. Analysis used up to 11,914,699 variants from the Haplotype Reference Consortium panel, focusing on samples of European ancestry. After selecting 407,746 individuals of white British ancestry and applying quality filters via PLINK2 [36] that included: a minor allele frequency of ≥ 1%, a Hardy–Weinberg equilibrium test not exceeding *P* = 1 × 10^− 15^, a genotyping rate above 99%, not present in low-complexity regions, not involved in inter-chromosomal LD and LD pruning using a R^2^ threshold of 0.9 with a window size of 1,000 markers and a step size of 100 markers, up to 471,762 genotyped SNPs were retained for further analysis [35]. The SS data utilized in this study encompass both primary and secondary SS.

### Selection of IVs

Single nucleotide polymorphism (SNP)s from prior GWASs pertinent to circulating inflammatory cytokines were employed for MR assessment, adhering to a genome-wide significance threshold (*p* < 5 × 10^− 6^)). The chosen SNPs ensured no linkage disequilibrium (LD) with other SNPs, maintaining an $$ {r}^{2}$$ below 0.1 within a 500 kb clumping radius. Where SNPs surpassed the $$ {r}^{2}$$ = 0.1 threshold, only the SNP with the strongest association (smallest *P* value) with the cytokine was chosen. This selection approach aligns with methods traditionally utilized in preceding studies [37, 38]. To counter possible bias from subpar instruments, the $$ {R}^{2}$$ and F statistics for each SNP were ascertained using specified formulas:

$$ {R}^{2}=\frac{2\times {\beta }^{2}\times EAF\times (1-EAF)}{[2\times {\beta }^{2}\times EAF\times \left(1-EAF\right)+2\times {\left(se\right({\beta }^{2})}^{2}\times N\times EAF\times \left(1-EAF\right)]}$$

$$ F=\frac{N-k-1}{k}\times \frac{{R}^{2}}{1-{R}^{2}}$$

Here, β, EAF, se(β), N, and k denote genetic variant effect size, effect allele frequency, standard error of this effect size, exposure sample size, and SNP count, respectively. SNPs with F below 10 were discarded. Procedures extracted and harmonized outcome-associated SNPs, excluding correlated (*p* < 5 × 10^− 6^), palindromic, and allele inconsistent SNPs, leading to an MR study on cytokines with over two SNPs [39].

### Statistical analysis

To ascertain the causal relationship between circulating inflammatory cytokines and SS, a multi-faceted methodological approach was adopted in this research. This included the utilization of inverse variance weighted (IVW), MR-Egger regression, MR-Egger intercept, weighted median and weighted mode strategies. The aggregate effect of circulating inflammatory cytokines on SS was delineated via a meta-analysis technique, amalgamating Wald estimates for each SNP through the IVW method [17]. In endorsing significant results, heterogeneity and horizontal pleiotropy tests were conducted employing meta-analytical techniques, encompassing the modified Cochran Q statistic calculated for IVW and MR-Egger estimates [40]. To mitigate the heterogeneity impact attributed to a singular SNP, a leave-one-out analysis was enacted, systematically excluding one SNP at a time. Under the absence of horizontal pleiotropy, IVW results remain impartial. The MR-Egger regression, predicated on the InSIDE assumption that instrument strength bears no correlation to a direct effect, evaluates pleiotropy through its intercept term. A zero intercept term in x aligns with IVW results, denoting an absence of horizontal pleiotropy [14]. The weighted median technique facilitates accurate causal deductions, even with up to 50% of the IVs deemed invalid [31]. When the InSIDE assumption faces challenges, the weighted mode estimate yields augmented power, diminished bias, and a reduced type I error rate for MR-Egger regression [41]. The evaluations were facilitated by the “TwoSampleMR” R packages, version 4.1.3 [42].

## Result

### Selection of instrumental variables

Upon stringent quality control of IVs, 91 cytokines were included in the MR analysis (Supplementary Table S1). These IVs comprised SNPs ranging from 9 to 27 (with Axin-1 genetically represented by 9 SNPs and tumor necrosis factor receptor superfamily member 9 levels having the highest representation with 27 SNPs). No evidence of pleiotropic effects was detected by the MR regression intercept test (*P* > 0.05). The F-statistics of IVs ranged between 20.90 and 4995.16, all largely > 10, indicating no evidence of weak instrument bias (Table S2).

### Causal effects of cytokines on SS

**Fig. 2:**
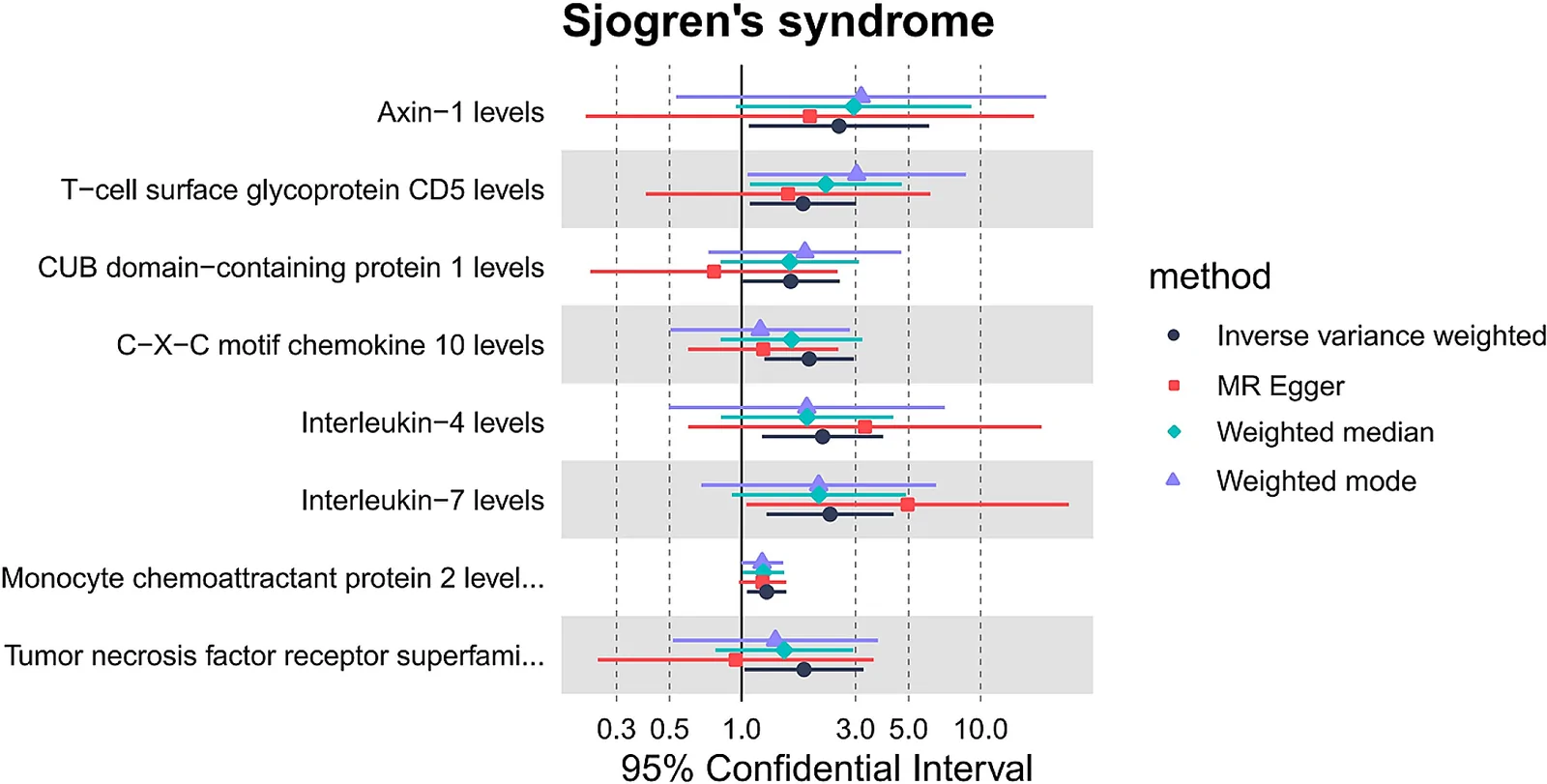
Forest plot of MR analysis. Forest plot to visualize the causal effect of cytokines on the risk of SS risk factors by Inverse variance weighted, MR Egger regression, Weighted median and Weighted mode method. Axin-1 levels, T-cell surface glycoprotein CD5 levels, CUDP1 levels, CXCL10 levels, IL-4 levels, IL-7 levels, MCP-2 levels and TNFRSF9. This plot shows these cytokines may increase the risk of SS.

Subsequent IVW analysis, paired with supplemental and sensitivity evaluations, pinpointed 8 cytokines that satisfied the stringent selection criteria as potential candidates (Fig. 2) (Table 1). The selected risk factors were as followed: Axin-1 levels, T-cell surface glycoprotein CD5 (T-cell CD5) levels, CUB domain-containing protein 1 (CUDP1) levels, CXCL10 levels, IL-4 levels, IL-7 levels, Monocyte chemoattractant protein 2 (MCP-2) levels, Tumor necrosis factor receptor superfamily member 9 (TNFRSF9) levels. All selected cytokines are positive association with SS: Axin-1 levels (OR 2.56, 95% CI 1.07 to 6.10, *p* = 0.034), T-cell CD5 levels (OR 1.81, 95% CI 1.08 to 3.02, *p* = 0.024), CUDP1 levels (OR 1.61, 95% CI 1.00 to 2.58, *p* = 0.049), CXCL10 levels (OR 1.92, 95% CI 1.25 to 2.95, *p* = 0.003), IL-4 levels (OR 2.18, 95% CI 1.22 to 3.91, *p* = 0.009), IL-7 levels (OR 2.35, 95% CI 1.27 to 4.33, *p* = 0.006), MCP-2 levels (OR 1.27, 95% CI 1.05 to 1.54, *p* = 0.011), TNFRSF9 (OR 1.83, 95% CI 1.03 to 3.24, *p* = 0.040). This suggests that these cytokines may increase the risk of SS.

**Table 1:** The IVW results of 8 cytokines

Exposure	NSNP	B	OR (95% CI)	*P* value
Axin-1 levels	8	0.94	2.56 (1.07–6.1)	0.034
TNFRSF9 levels	21	0.60	1.83 (1.03–3.24)	0.040
CDCP1 levels	18	0.47	1.61 (1.0-2.58)	0.049
CXCL10 levels	21	0.65	1.92 (1.25–2.95)	0.003
IL-4 levels	4	0.78	2.18 (1.22–3.91)	0.009
IL-7 levels	14	0.85	2.35 (1.27–4.33)	0.006
MCP-2 levels	18	0.24	1.27 (1.05–1.54)	0.011
T-cell CD5 levels	19	0.59	1.81 (1.08–3.02)	0.024

**Fig. 3:**
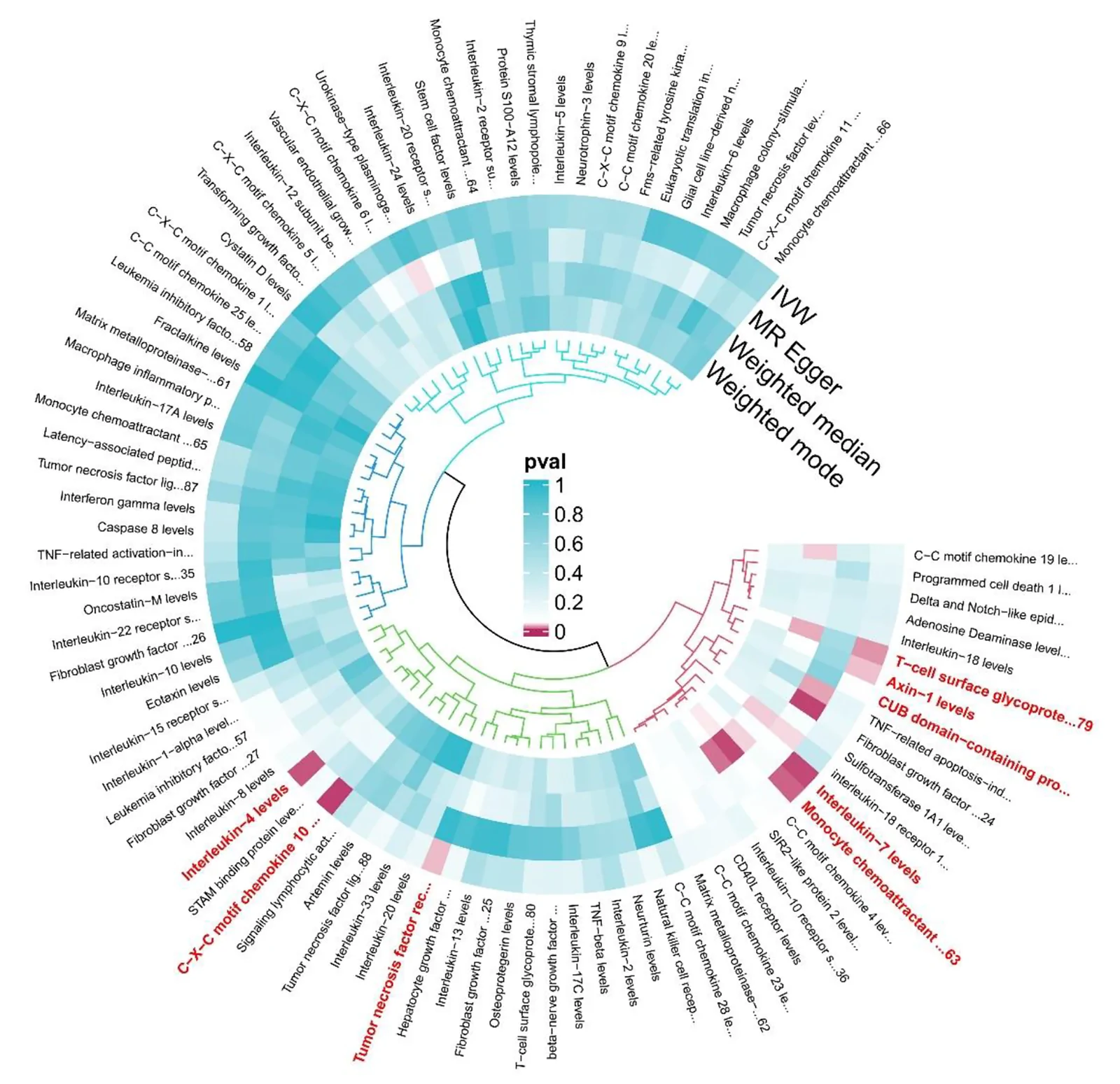
Preliminary MR analyses for the associations between inflammatory cytokines and the risk of SS. The circle from the outer to the inner represented the IVW, MR-Egger regression, weighted median, and weighted mode, respectively. The shades of color were reflections of the magnitude of the *p*-value as the label inside the circle. (MR Egger, MR Egger regression; IVW, inverse variance-weighted.)

**Fig. 4:**
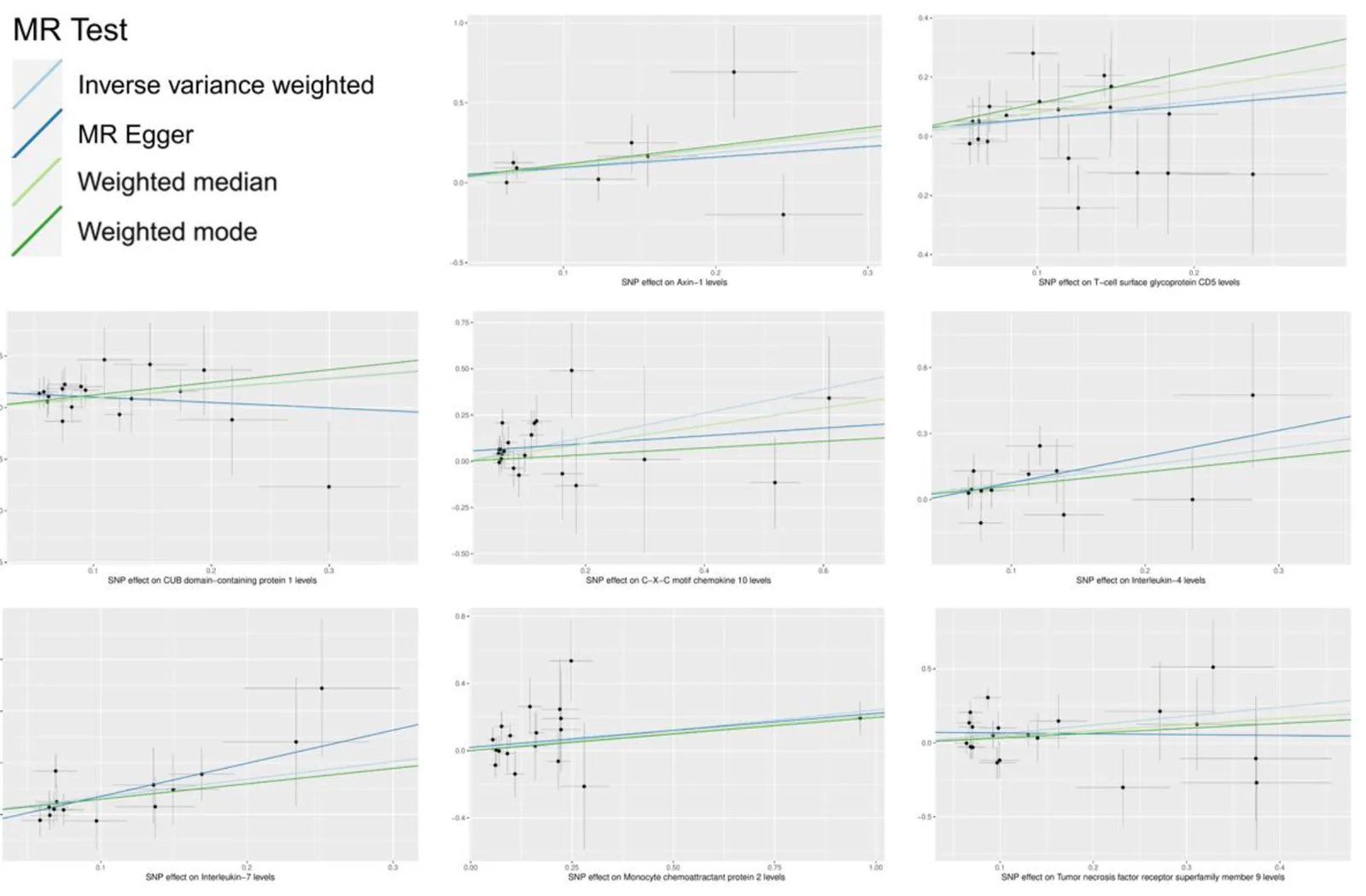
Scatter plots of MR analysis. Analyses were conducted using IVW, MR Egger, Weighted median and Weighted mode. The slope of each line corresponding to the estimated MR effect per method

In summation, IVW-derived estimates were significant (*p* < 0.05), and there was consistency in direction and magnitude across IVW, MR-Egger, weighted median, and weighted mode estimates (Fig. 3) (Supplementary Table S3). Scatter plots for identified cytokines across various tests are displayed on Fig. 4. Both the Cochran Q test (*p* > 0.05) and the MR-Egger intercept test (*p* > 0.05) strongly supported the lack of heterogeneity and pleiotropy (Table 2). Leave-one-out analysis affirmed that no individual SNP introduced bias into the MR estimation (SupplementaryFigure S1). The funnel plots were showed on SupplementaryFigure S2.

**Table 2:** Heterogeneity and horizontal pleiotropy of MR

Exposure	*P* value (Cochran Q)	*P* value (MR intercept)
Axin1 levels	0.318	0.787
CXCL10 levels	0.478	0.152
IL-4 levels	0.548	0.628
IL-7 levels	0.829	0.326
T-cell CD5 levels	0.273	0.826
CUDP1 levels	0.9	0.204
MCP-2 levels	0.631	0.553
TNFRSF9 levels	0.051	0.296

## Discussion

SS is an autoimmune condition that primarily affects the salivary and lacrimal glands, leading to conditions like xerostomia (dry mouth) and xerophthalmia (dry eyes). The intricate dance of cytokines, receptors, and immune cells contributes to the onset and progression of this disease. Our MR experiment has shed light on several key players in this scenario, and integrating the findings from various studies paints a richer picture of the SS.

The results indicate a strong association between increased levels of CXCL10 and the risk of an autoimmune response (OR 1.92, *p* = 0.003). The chemokine CXCL10 is known to exert its functions through its receptor CXCR3 [43]. This binding plays a pivotal role in the pathogenesis of a myriad of autoimmune diseases, ranging from organ-specific diseases such as type 1 diabetes and Graves’ disease to systemic conditions like rheumatoid arthritis and SS [44]. A mechanism through which this occurs is that the secretion of CXCL10 by various immune cells, including CD4+, CD8+, NK, and NK-T cells, is dependent on IFN-γ, which itself is mediated by the interleukin-12 cytokine family [45]. It is interesting to note that high levels of CXCL10 in peripheral fluids act as a marker of host immune response, predominantly by Th1 orientated T-cells [46]. This Th1 response in tissues potentially leads to the enhanced production of IFN-γ and tumor necrosis factor-α, stimulating further CXCL10 secretion [47]. This amplification feedback loop thus perpetuates the autoimmune process. Given these insights, CXCL10 could indeed be a novel therapeutic target for autoimmune diseases.

CD5, a T-cell surface glycoprotein, exhibited an association with increased autoimmune risk (OR 1.81, *p* = 0.024). Delving into the literature, CD5 seems to play an intriguing role in SS [48]. In patients with primary SS, a reduced expression and function of CD5 molecule on peripheral blood lymphocytes were observed [49]. Additionally, the ratio of CD5 + to CD3 + lymphocytes was significantly lower in these patients compared to normal subjects, highlighting a CD5 deficiency [48]. This reduction has potential implications, as CD5 could be involved in intracellular signaling defects in primary SS [49]. This conclusion is further solidified by observations where a correction in the CD5 lymphocyte abnormality was associated with clinical remission in some SS patients. Additionally, studies on lip biopsy specimens from SS patients also revealed that a significant portion of cellular infiltrates expressed the CD5 molecule [48, 50]. This further accentuates the importance of CD5 in the disease pathology of SS.

The role of IL-7, another interleukin, in SS cannot be understated. Our results show a noteworthy association between elevated Interleukin-7 levels and the disease (OR 2.35, *p* = 0.006). This is consistent with the findings from a single-center study, where primary SS patients exhibited higher serum IL-7 levels [25]. The systematic scoping review further emphasized the potential of IL-7 as a biomarker for monitoring primary SS activity [51]. Elevated IL-7 levels, mainly from salivary glands, could be pivotal in primary SS immunopathology. Transitioning to IL-4 (OR 2.18, *p* = 0.009), research in NOD/LtJ mice linked to SS found increased B cell infiltration and salivary gland apoptosis. Leptin treatment led to IL-4 secretion from B cells, hinting at the Leptin/OB-R pathway’s role in promoting SGEC apoptosis [52]. Studies using IL-4 gene knockout NOD mice highlighted IL-4’s essential role in autoimmune xerostomia development. While the exact mechanism remains uncertain, IL-4’s impact exists [53].

Our study also identified a significant association between elevated TNFRSF9 levels and SS (OR 1.83, *p* = 0.04). But there is no previous study discovered TNFRSF9’s relation to SS. However, CD137 (4-1BB), a surface glycoprotein that belongs to TNFRSF9 has reported having relation to SS. This finding is particularly noteworthy because 4-1BB is a costimulatory receptor that has been shown to modulate T cell responses. A study on the NOD model of SS highlighted that activation of 4-1BB could impede the development of sialadenitis by modulating various immune cell types and their associated cytokines [54, 55]. Previous studies in MRL-Faslpr mice also demonstrated that the expression of costimulatory molecules GITRL and 4-1BBL in salivary glands was significantly correlated with the severity of autoimmune sialadenitis [55]. Our findings are in line with these observations and extend them by providing genetic evidence may implicating TNFRSF9 in the pathogenesis of SS.

Our study also discovered new cytokine related to SS. Axin-1 levels, MCP-2 levels and CDCP1 levels have been found having a significant positive correlation between the levels of the risk of SS, which is not reported in previous studies. This may suggest a diverse role of the cytokine in the onset and progression of SS. The novel findings may contribute to a more comprehensive understanding of the circulating inflammatory cytokine of SS and offer new potential biomarkers for the early diagnosis and prevention of SS.

Several limitations exist within this study. Firstly, the exposure of interest at the genome-wide level had a restricted number of SNPs. This was addressed by applying slightly relaxed thresholds for the MR analysis, mirroring practices in previous research. Nonetheless, with F-statistic values for the chosen SNPs surpassing 10, the robustness of our IVs is indicated. Secondly, our MR analysis exclusively utilized GWAS data from European ancestry individuals, limiting ethnic variability. Consequently, the applicability of these findings to diverse populations necessitates further investigation and validation. Thirdly, the number of cytokines included in this study is limited. Prior genomic studies have identified associations in SS with molecules of the human leukocyte antigen complex and with transcription factors related to the IFN signature [56–58]. Therefore, analysis about broader array of cytokines is needed in the future. Fourthly, the SS data utilized in this study encompass both primary and secondary SS. Material from primary SS or secondary SS may influence genomic and proteomic analysis. Carballeda J et al.’s study reported primary SS patients exhibited a greater number of CD4(+)/IL-17 A(+) and IL-19(+) T cells but a lower percentage of IL-24(+) cells (*P* < 0.05) than secondary SS [59]. Subsequent analyses that categorize SS into these subtypes may uncover distinct impacts of cytokines on primary versus secondary SS. Fifthly, the MR estimation’s precision is inherently tied to sample size, emphasizing the need to augment the sample size for result validation. While MR analysis sheds light on disease etiology, it’s imperative to corroborate our findings through rigorous RCTs and foundational research before clinical integration.

## Conclusion

our study, conducted through MR, identified various inflammatory cytokines associated with SS risk, validating some previous research results and offering some new potential biomarkers for SS. However, these findings necessitate further research for validation and exploration of their precise role in the onset and progression of SS.

## Electronic supplementary material

Below is the link to the electronic supplementary material.

Supplementary Material 1

Supplementary Material 2

Supplementary Material 3

Supplementary Material 4

Supplementary Material 5

## Data Availability

We have annotated the article with the source of all original data, please contact the original authors for access if needed. The results of this study can be obtained by contacting the corresponding author.

## Abbreviations

SS: Sjogren’s Syndrome
MR: Mendelian Randomization
RCT: Randomized controlled trial
IV: Instrumental variable
IVW: Inverse variance weighted
STROBE-MR: Strengthening the Reporting of Observational Studies in Epidemiology using MR
LD: Linkage disequilibrium
CUDP1: CUB domain-containing protein 1
CXCL10: C-X-C motif chemokine 10
IL: Interleukin
MCP-2: Monocyte chemoattractant protein 2
TNFRSF9: Tumor necrosis factor receptor superfamily member 9 levels
SNP: Single nucleotide polymorphism
IFN: interferon
